# Reciprocity between State Boards of Medical Examiners

**Published:** 1895-11

**Authors:** 


### RECIPROCITY BETWEEN STATE BOARDS OF MEDICAL EXAMINERS.

WE BELIEVE the day is at hand (*Va. Med. Monthly*) when it is safe to advocate the laws of reciprocity between states having boards of examiners who adopt the same standard of requirements. This is a matter which, from the very nature of affairs, must very soon engage the serious attention of the profession. If the state laws require that all applicants for examination shall hold the diplomas of reputable medical colleges demanding a three or four years' course, as seems to be the tendency of the day, and if the law of the state in which the graduate may begin practice requires that he shall first be examined by the state board of medical examiners to test his qualifications, then it appears altogether reasonable that the licentiate of one state board may receive the certificate of another state board of equal demand as a sufficient test of qualifications to practise in that state. It looks a little hard for the young graduate who has already spent three or four years in preparing himself for his diploma, and who has since graduation satisfactorily stood before his state board, to be kept constantly preparing for examinations if he wishes to change his location from one state to another. It is not generally known that even between France and Germany there is a territory extending for fifteen miles on either side of the boundary line in which medical men who have the license of their nation may discharge their professional duties without molestation. "There is also an understanding between Quebec and Manitoba, so that the licenses granted by the boards of these respective provinces allow candidates to practise in either without further trouble.'' Quebec and New Brunswick had a similar understanding, but some irregularities occurred and they no longer reciprocate.

Then, also, there are cases in which the requirement of examination of thoroughly able practitioners—recognised as such everywhere they are known—may very seriously embarrass the interests of a state. Thus, suppose a surgeon residing in another state—recognised the world over as an educated, competent, able one—were needed as professor of surgery in one or the other of Virginia's medical colleges? Whatever might be the inducement to accept, it is natural that he should be disposed to decline when he knows that he will, at his mature age and ripe experience as a surgeon, have to undertake again the study of the technicalities of chemistry, physiology and the like, in order that he may comply with the laws of the state in which he desired to go. Some law permitting reciprocity or the form of courtesy just intimated should be adopted in each state.

It is not necessary, nor do we advocate, that the statute of any state should compel its state board to adopt the certificate of another state board; but what is needed is a change in the laws which would *allow* a given state board to receive the certificate of another state board as sufficient evidence on the part of the lawful holder that he is qualified to practise without further examination.

